# Comparison of clinical characteristics and outcomes of hospitalized patients with seasonal coronavirus infection and COVID-19: a retrospective cohort study

**DOI:** 10.1186/s12879-022-07555-4

**Published:** 2022-07-15

**Authors:** Guillermo Rodriguez-Nava, Goar Egoryan, Tianyu Dong, Qishuo Zhang, Elise Hyser, Bidhya Poudel, Maria Adriana Yanez-Bello, Daniela Patricia Trelles-Garcia, Chul Won Chung, Bimatshu Pyakuryal, Taraz Imani-Ramos, Valeria Patricia Trelles-Garcia, Daniel Sebastian Bustamante-Soliz, Jonathan J. Stake

**Affiliations:** 1 Department of Internal Medicine, AMITA Health Saint Francis Hospital, 355 Ridge Ave, Evanston, IL 60202 USA; 2 Department of Internal Medicine, AMITA Health Saint Joseph Hospital, Chicago, IL USA; 3 Department of Internal Medicine, John H. Stroger Jr. Hospital of Cook County, Chicago, IL USA; 4 Facultad de Ciencias Medicas de La Universidad de Cuenca, Cuenca, Ecuador; 5 Department of Infection Prevention, AMITA Health Saint Francis Hospital, Evanston, IL USA

**Keywords:** COVID-19, Human coronavirus, Seasonal coronavirus, SARS, MERS

## Abstract

**Background:**

Unlike SARS-CoV and MERS-C0V, SARS-CoV-2 has the potential to become a recurrent seasonal infection; hence, it is essential to compare the clinical spectrum of COVID-19 to the existent endemic coronaviruses. We conducted a retrospective cohort study of hospitalized patients with seasonal coronavirus (sCoV) infection and COVID-19 to compare their clinical characteristics and outcomes.

**Methods:**

A total of 190 patients hospitalized with any documented respiratory tract infection and a positive respiratory viral panel for sCoV from January 1, 2011, to March 31, 2020, were included. Those patients were compared with 190 hospitalized adult patients with molecularly confirmed symptomatic COVID-19 admitted from March 1, 2020, to May 25, 2020.

**Results:**

Among 190 patients with sCoV infection, the Human Coronavirus-OC93 was the most common coronavirus with 47.4% of the cases. When comparing demographics and baseline characteristics, both groups were of similar age (sCoV: 74 years vs. COVID-19: 69 years) and presented similar proportions of two or more comorbidities (sCoV: 85.8% vs. COVID-19: 81.6%). More patients with COVID-19 presented with severe disease (78.4% vs. 67.9%), sepsis (36.3% vs. 20.5%), and developed ARDS (15.8% vs. 2.6%) compared to patients with sCoV infection. Patients with COVID-19 had an almost fourfold increased risk of in-hospital death than patients with sCoV infection (OR 3.86, CI 1.99–7.49; p < .001).

**Conclusion:**

Hospitalized patients with COVID-19 had similar demographics and baseline characteristics to hospitalized patients with sCoV infection; however, patients with COVID-19 presented with higher disease severity, had a higher case-fatality rate, and increased risk of death than patients with sCoV. Clinical findings alone may not help confirm or exclude the diagnosis of COVID-19 during high acute respiratory illness seasons. The respiratory multiplex panel by PCR that includes SARS-CoV-2 in conjunction with local epidemiological data may be a valuable tool to assist clinicians with management decisions.

## Background

Coronaviruses are large, enveloped, single-stranded RNA viruses found in humans and other animals, such as dogs, cats, bats, chickens, cattle, pigs, and birds. These viruses have the potential to cause respiratory, enteric, hepatic, and neurologic diseases. The most common coronaviruses in clinical practice are 229E, OC43, NL63, and HKU1, which typically cause common cold symptoms in immunocompetent individuals and contribute 15% to 30% of common cold cases [1, 2]. Two other strains, the severe acute respiratory syndrome coronavirus (SARS-CoV) and the Middle East respiratory syndrome coronavirus (MERS-CoV), are associated with severe respiratory disease and are responsible for the first significant coronavirus outbreaks [2, 3]. On December 21, 2019, a novel coronavirus was identified in hospitalized patients with pneumonia in Wuhan, China. Genetic analysis revealed that this novel coronavirus fits into the genus betacoronavirus. Further phylogenetic analysis showed that the SARS-CoV-2 virus belongs to the subgenus Sarbecovirus and that is more similar to two bat-derived coronavirus strains, bat-SL-CoVZC45 and bat-SL-CoVZXC21, than to known human-infecting coronaviruses, including SARS-CoV [3, 4].

Because seasonal coronaviruses are regarded as mild upper respiratory pathogens with a known peak prevalence during December–March each year in the U.S. (coinciding with the winter respiratory virus season), molecular testing is not frequently performed in the clinical outpatient practice, and it is reserved for surveillance purposes [5]. However, because of the increased availability of molecular test methods and the adoption of sCoV testing as part of routine multiplex diagnostic screens, particularly for patients with severe respiratory illness or admitted to critical care units where a precise microbiologic diagnosis is more clinically relevant, it is now possible to recognize and characterize the associated disease spectrum of severe sCoV infections and compare it to that of COVID-19 [5, 6]. The clinical presentation, diagnostics, and outcomes of patients with COVID-19 have been well described in multiple case series and cohort studies [7–10] and compared to hospitalized patients with other respiratory viruses [11–14]. Nevertheless, there is limited data on how COVID-19 compares clinically to seasonal coronaviruses (sCoV). Unlike SARS-CoV and MERS-CoV, SARS-CoV-2 carries the potential to become a recurrent seasonal infection; hence, it is essential to compare the clinical spectrum of COVID-19 to the existent endemic coronaviruses in an attempt to help clinicians distinguish both entities during potential co-circulation throughout winter seasons and guide further management [5, 15, 16]. Thus, this study compares the clinical characteristics, course, and outcomes of hospitalized patients with COVID-19 with hospitalized patients with sCoV infection.

## Methods

### Design, setting, and participants

This cross-sectional retrospective cohort study included 380 hospitalized adult patients (18 years or older) with sCoV or COVID19 across four AMITA Health hospitals located in the Chicago metropolitan area. A total of 190 patients hospitalized with pneumonia (ICD-10-CM Code J18.9), upper respiratory tract infection (ICD-10-CM Code J06.9) or lower respiratory tract infection (ICD-10-CM Code J22), and a positive respiratory viral panel (BioFire® FilmArray Respiratory Panel) for sCoV from January 1, 2011, to March 31, 2020, were identified by the Electronic Health Records department and thus, no sample size calculation was performed. Those patients were compared with 190 patients randomly selected from a de-identified dataset that included 313 hospitalized adult patients with molecularly confirmed new-onset symptomatic COVID-19 (Abbott™ RealTi*me*™ SARS-CoV-2 assay or Abbott™ ID NOW COVID-19™ assay) admitted from March 1, 2020, to May 25, 2020.

### Definitions

Respiratory failure was defined as room air oxygen saturation less than or equal to 90% or using any means of supplemental oxygen associated with shortness of breath. Sepsis and septic shock were defined according to the 2016 Third International Consensus Definition for Sepsis and Septic Shock [17]. Acute kidney injury (AKI) was diagnosed according to the KDIGO clinical practice guidelines [18], and acute respiratory distress syndrome (ARDS) was diagnosed according to the Berlin Definition [19]. Troponin leak was defined as non-ACS cardiac troponin elevation above reference range levels [20]. The severity of COVID-19 illness and sCoV infections was defined and unified according to the National Institutes of Health guidelines for the management of COVID-19 [21]. Other definitions include: residents of long-term care facilities as residents of group, board and care homes, assisted living facilities, nursing homes, or continuing care retirement communities; neurocognitive impairment as any dementia, Parkinson’s disease with cognitive impairment, intellectual disability, or cerebral palsy; altered mental status as any alteration in alertness, orientation or level of consciousness; immunosuppression as patients on daily dose ≥ 20 mg of prednisone or equivalent, active chemotherapy, immunotherapy, immunomodulators (immunosuppressants), or patients diagnosed with any hematological neoplasia.

### Data collection

Clinical data were manually extracted and collected by the investigators via retrospective chart review from an electronic medical record system (Epic). Information collected included demographic data, medical history, underlying comorbidities, symptoms, signs, laboratory findings, imaging studies, treatment measures, survival to hospital discharge (survivors), and in-hospital death or referral to hospice (nonsurvivors). A 10% random sample was re-abstracted to ascertain agreement and monitor calibration. We calculated a Cohen’s kappa for each categorical variable and intraclass correlation coefficient for continuous variables included in the analysis. The mean (SD) Cohen’s kappa for categorical variables was 0.85 (0.15), with a percentage agreement of 94%, indicating a strong level of interrater agreement. The mean intraclass correlation coefficient for continuous variables was 0.94 (0.08), indicating excellent interrater reliability.

The study was approved by the Institutional Review Board of AMITA Health System (2021-0180-02). The Ethics Commission waived the requirement for informed consent, given that this research involves no more than minimal risk to participants.

### Statistical analysis

Descriptive statistics were used to summarize the data; categorical variables were described as frequency and percentages, and continuous variables were described using median and interquartile range (IQR) values. Non-normal distribution was confirmed with the Shapiro–Wilk test. We used the Mann–Whitney U test, Chi-squared test, or Fisher exact test to compare differences between patients with sCoV infection and COVID-19 when appropriate. An exploratory unconditional multivariable logistic regression model with generalized estimating equations with exchangeable correlation structure correcting standard error estimates for site-level clustering was used to assess differences in case-fatality between patients with sCoV infection and participants with COVID-19 [22], adjusting for age, residence (home or long-term care facility [LTCF]), do-not-resuscitate/do-not-intubate (DNR/DNI) status and quick Sequential Organ Failure Assessment (qSOFA) score. We opted to fit these variables into the model based on clinical knowledge and previous literature. A two-sided alfa of less than 0.05 was considered statistically significant.

## Results

### Demographics and baseline characteristics

**Fig. 1:**
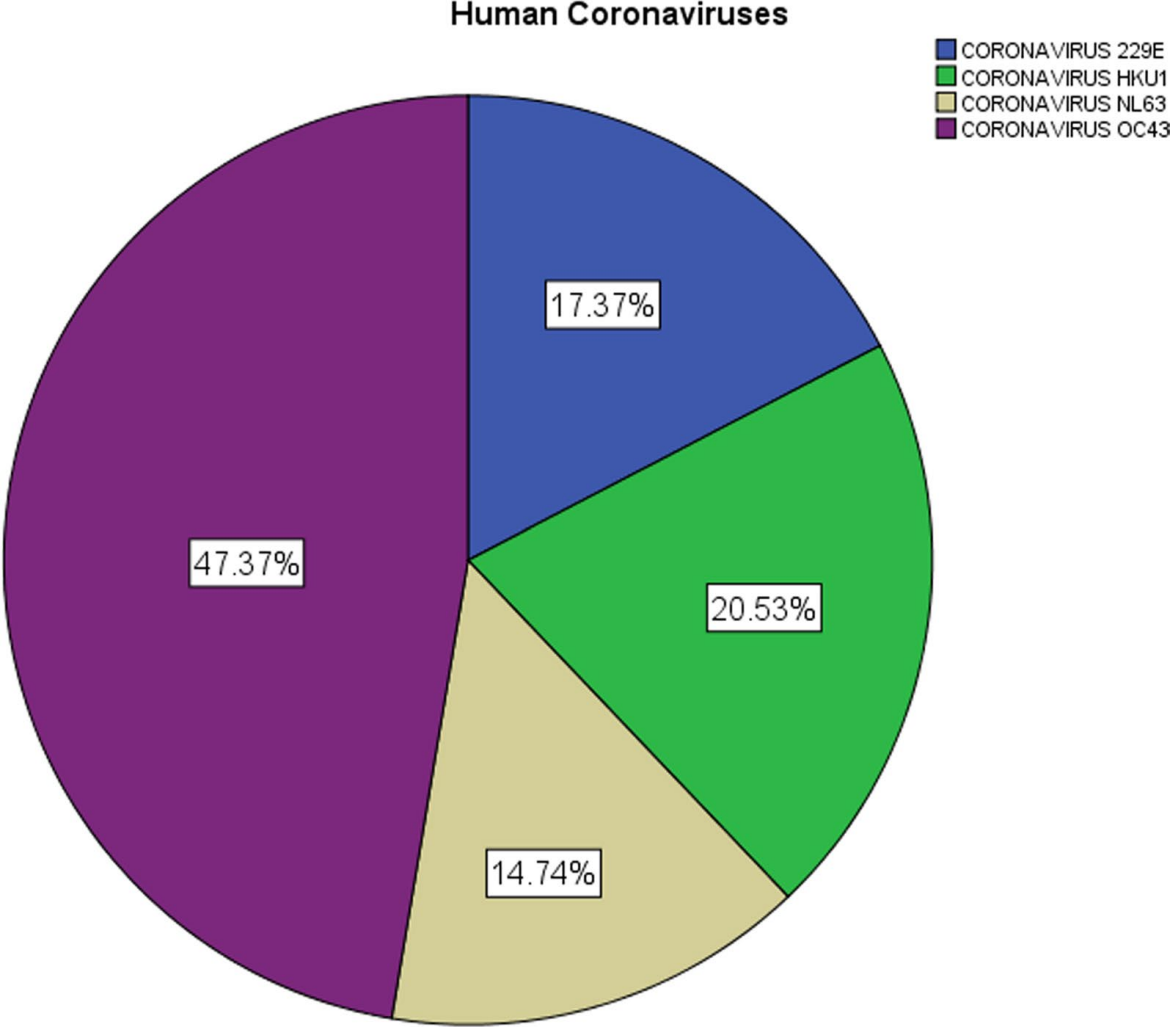
Distribution of human coronavirus species among 190 patients with seasonal coronavirus infection

The median age of the base cohort was 72 years (IQR, 59.0–83.0 years; range 21–98 years) and 203 (53.4%) were male. Among patients with sCoV infection, the Human Coronavirus (HCoV)-OC93 was the most common coronavirus with 47.4% of the cases, followed by HCoV-HKU1 (20.5%), HCoV-229E (17.4%), and HCoV-NL63 (14.7%) (Fig. 1). Baseline characteristics, disease severity, and inpatient case-fatality rates were not significantly different between each sCoV, except for a significantly higher rate of inpatients with CoV-HKU1 and a history of COPD and a significantly higher rate of patients with CoV-229E who required IMV (Table 1).

**Table 1:** Comparison between inpatients with human seasonal coronaviruses

	CoV-229E (N = 33)	CoV-HKU1 (N = 39)	CoV-NL63 (N = 28)	CoV-OC43 (N = 90)	P-value
Age in years^a^	72 (63–81.5)	69 (55–82)	75.5 (56.5–84)	75.5 (64.5–87.25)	.164
Male	18 (54.5%)	22 (56.4%)	12 (42.9%)	37 (41.1%)	.310
White (vs. all other)	25 (75.8%)	19 (48.7%)	19 (67.9%)	56 (62.2%)	.112
Home (vs. SNF)	22 (66.7%)	25 (64.1%)	17 (60.7%)	57 (63.3%)	.971
Two or more comorbidities	27 (81.8%)	36 (92.3%)	25 (89.3%)	75 (83.3%)	.473
Obesity	12 (36.4%)	14 (35.9%)	8 (28.6%)	25 (27.8%)	.707
COPD	8 (24.2%)	20 (51.3%)^b^	8 (28.6%)	32 (35.6%)	.085
Abnormal CXR	26 (78.8%)	28 (71.8%)	21 (75%)	65 (72.2%)	.887
Severe illness	26 (78.8%)	25 (64.1%)	20 (71.4%)	58 (64.4%)	.439
ICU	14 (42.4%)	13 (33.3%)	14 (50%)	26 (28.9%)	.167
IMV	9 (27.3%)^c^	6 (15.4%)	2 (7.1%)	10 (11.1%)	.089
Nonsurvivors	4 (12.1%)	2 (5.1%)	5 (17.9%)	11 (11.2%)	.439

^a^Shapiro–Wilk normality test results showed deviation from a normal distribution

^b^P-value obtained with a Bonferroni Chi-Square residual analysis: p = .023

^c^P-value obtained with a Bonferroni Chi-Square residual analysis: p = .018

When comparing demographics and baseline characteristics between inpatients with sCoV and COVID-19, both groups were of similar age, more patients with sCoV infection were female, White, and admitted from home, while patients with COVID-19 were more likely to be male and admitted from an LTCF. Of note, more patients with COVID-19 were admitted with DNR/DNI orders (Table 2). The proportion of patients with two or more comorbidities, obesity and a history of smoking was not significantly different between patients with sCoV infection and COVID-19. However, patients with sCoV infection presented higher rates of cardiovascular disease, history of malignancies, COPD or asthma, and immunodeficiency, whereas patients with COVID-19 presented higher rates of diabetes and neurocognitive disorders (Table 2).

**Table 2:** Differences in baseline characteristics and clinical presentation between seasonal coronaviruses and COVID-19 inpatients

	sCoV (N = 190)	COVID-19 (N = 190)	P-value
Demographics			
Age in years^a^	74 (59–84)	69 (59–82)	.081
Male (vs. female)	89 (46.8%)	114 (60%)	.010
White (vs. all other)	119 (62.6%)	76 (40%)	< .001
LTCF (vs. home)	69 (36.3%)	123 (64.7%)	< .001
DNR/DNI	53 (27.9%)	74 (38.9%)	.022
Two or more comorbidities	163 (85.8%)	155 (81.6%)	.267
Cardiovascular	89 (46.8%)	65 (34.2%)	.012
Obesity	59 (31.1%)	55 (28.9%)	.654
Diabetes	73 (38.4%)	92 (48.4%)	.049
Malignant disease or mass	42 (22.1%)	20 (10.5%)	.002
Neurocognitive disorder	51 (26.8%)	69 (36.3%)	.047
COPD or asthma	68 (35.8%)	42 (22.1%)	.003
HIV or other immunodeficiency	23 (12.1%)	3 (1.6%)	< .001
Never smoker (vs. former or current)	103 (54.2%)	110 (57.9%)	.469
Symptoms			
Fever	96 (50.5%)	117 (61.6%)	.030
Chills	46 (24.2%)	16 (8.4%)	< .001
Cough	143 (75.3%)	103 (54.2%)	< .001
Shortness of breath	143 (75.3%)	130 (68.4%)	.138
Anosmia	1 (0.5%)	7 (3.7%)	.032
Diarrhea	7 (3.7%)	25 (13.2%)	.001
Signs			
Altered mental status	43 (22.6%)	88 (46.3%)	< .001
Temperature (°C)^a^	37.1 (36.7–38.1)	37.8 (37–38.625)	< .001
Lowest SpO2 in the ED (%)^a^	93 (88–95)	93 (88–95)	.680
Systolic blood pressure (mmHg)^a^	132 (114–160)	120.5 (102–139.25)	.014
Heart rate (bpm)^a^	100.5 (86–116.25)	97 (81–111)	.259
Respiratory rate (rpm)^a^	22 (20–28)	22 (20–28)	.757
Labs^a^			
White blood cells (4.0–11.0, × 109/L)	10.75 (7.3–15.025)	7.9 (5.575–11.70)	< .001
Lymphocyte count (0.6–3.4, × 109/L)	1 (0.6–1.625)	0.9 (0.6–1.3)	.148
Hemoglobin (12.0–15.3, g/dL)	12.1 (10.675–13.60)	12.8 (11.4–14.2)	.010
Platelets (150–450, × 109/L)	216.5 (162.5–292)	206 (160.5–277.5)	.473
Serum creatinine (0.6–1.3, mg/dL)	1.01 (0.77–1.43)	1.31 (0.93–2.17)	< .001
Blood urea nitrogen (7–25, mg/dL)	23 (15–36.25)	28 (17–46)	.010
Lactic acid (0.7–2.0, mmol/L)	1.8 (1.3–2.75)	1.7 (1.2–2.4)	.621
Chest X-rays			
No acute findings	50 (25.3%)	28 (14.7%)	.009
Unilateral opacities	82 (43.2%)	45 (23.7%)	< .001
Bilateral opacities^c^	49 (25.8%)	92 (48.4%)	< .001
Diffuse opacities^c^	9 (4.7%)	25 (13.2%)	.003

^a^Shapiro-Wilk normality test results showed deviation from a normal distribution

^b^Two or more co-existing comorbidities

^c^Bilateral opacities: Lung infiltrates present in both lung fields but < 50%; Diffuse opacities: Lung infiltrates > 50% in both lung fields

COPD: chronic obstructive pulmonary disease; COVID-19: Coronavirus Disease 2019; DNR/DNI: do-not-intubate and do-not-resuscitate; ED: emergency department; HIV: human immunodeficiency virus; LTCF: long-term care facility; sCoV: seasonal coronavirus; SpO2: peripheral oxygen saturation; VTE: venous thromboembolism

### Clinical presentation and interventions

Upon presentation to the hospital, more patients with sCoV infection reported chills and cough, while more patients with COVID-19 reported fever, anosmia, and diarrhea. The rates of shortness of breath were not different between groups. Clinically, patients with COVID-19 presented higher rates of altered mental status, higher body temperature, and lower blood pressure than patients with sCoV infection (Table 2). Patients with sCoV infection presented a higher white blood count, while patients with COVID-19 presented higher serum creatinine levels and blood urea nitrogen (Table 2). Between patients with sCoV and COVID-19, there were no differences in the rates of leukopenia (white blood cells < 4.0 × 10^9^/L, 6.3% vs. 9.5%; p = 0.254), lymphopenia (lymphocyte count < 0.6 × 10^9^/L, 71.6% vs. 78.9%; p = 0.096), or thrombocytopenia (platelet count < 150 × 10^9^/L, 13.2 vs. 19.5%; p = 0.096). On imaging, a more significant proportion of patients with sCoV infection showed no acute findings or unilateral opacities, whereas more patients with COVID-19 were found to have bilateral or diffuse (Table 2).

With regards to interventions (Table 3), more patients with sCoV infection were placed on nonrebreather masks (12.1% vs. 6.3%) and noninvasive ventilation (13.2% vs. 1.1%) in the emergency department. On the other hand, more patients with COVID-19 were placed on high-flow nasal cannula (8.9% vs. 0.5%) and humidified high-flow system (3.7% vs. 0%). A similar proportion of patients required invasive mechanical ventilation (IMV) on presentation and later during the hospital stay. Both groups of patients with sCoV infection and COVID-19 were administered similar rates of steroids (45.3% vs. 43.7%) and antibiotics (95.8% vs. 91.1%). A larger proportion of patients with COVID-19 required vasopressors (16.8% vs. 10%), neuromuscular blockers (17.9% vs. 0.5%), and prone positioning (11.1% vs. 1.1%).

**Table 3:** Interventions, complications, and clinical outcomes among inpatients with seasonal coronaviruses and COVID-19

	sCoV (N = 190)	COVID-19 (N = 190)	P-value
Steroids	86 (45.3%)	83 (43.7%)	.757
Antibiotics	182 (95.8%)	175 (92.1%)	.132
Maximal respiratory support on presentation			.032
None	56 (29.5%)	56 (29.5%)	
Nasal cannula	95 (50%)	111 (58.4%)	
NIV	25 (13.2%)	9 (4.7%)	.004^b^
IMV	14 (7.4%)	14 (7.4%)	
Prone position	2 (1.1%)	21 (11.1%)	< .001
Neuromuscular blockade	1 (0.5%)	34 (17.9%)	< .001
Vasopressors	19 (10%)	32 (16.8%)	.050
Respiratory failure	134 (70.5%)	135 (71.1%)	.910
Sepsis			
SIRS	124 (65.3%)	120 (63.2%)	.669
qSOFA	39 (20.5%)	69 (36.3%)	.001
Septic shock	27 (14.2%)	38 (20%)	.134
ARDS	6 (2.6%)	38 (15.8%)	< .001
Acute kidney injury	48 (25.3%)	84 (44.2%)	< .001
Troponin leak	49 (25.8%)	55 (29.9%)	.373
Coinfection	49 (25.8%)	25 (13.2%)	.002
NIH severity			
Mild	14 (7.4%)	8 (4.2%)	.188
Moderate	47 (24.7%)	33 (17.4%)	.078
Severe	129 (67.9%)	149 (78.4%)	.021
Time from symptom onset to admission (days)^a^	3 (1–7)	2 (1–6)	.916
Hospital length of stay (days)^a^	5 (3–8)	7 (4–12)	.013
ICU admission	67 (35.3%)	61 (32.1%)	.515
IMV in total	27 (14.2%)	37 (19.5%)	.170
Successfully extubated	16/27 (59.3%)	13/37 (35.1%)	0.056
Successfully discharged from ICU	46/67 (73%)	26/61 (43.3%)	0.001
Onset to discharge (days)^a^	9 (6–13.75)	9.5 (7–16.75)	0.902
Onset to death (days)^a^	9 (5.75–15.25)	10 (6.75–16.25)	0.855
Case fatality rate	22 (11.6%)	66 (34.7%)	< .001

^a^Shapiro–Wilk normality test results showed deviation from a normal distribution

^b^P-value obtained with a Bonferroni Chi-Square residual analysis

ARDS: acute respiratory distress syndrome; COVID-19: Coronavirus Disease 2019; ICU: intensive care unit; IMV: invasive mechanical ventilation; NIV: noninvasive ventilation; qSOFA: quick Sequential Organic Failure Assessment; sCoV: seasonal coronavirus; SIRS: Systemic Inflammatory Response Syndrome

### Outcomes

Regarding inpatient outcomes (Table 3), patients with sCoV infection and COVID-19 developed similar respiratory failure rates. Patients with COVID-19 presented higher rates of sepsis, AKI, and ARDS. A higher number of individuals with sCoV were found to have co-infective organisms than individuals with COVID-19. Rates of mild and moderate illness were similar among both groups of patients on presentation, but significantly more patients with COVID-19 presented with severe disease. The time from symptom onset to discharge or death was not significantly different between patients with sCoV infection and COVID-19. Though, patients admitted with COVID-19 had a higher length of hospital stay than patients with sCoV. Rates of intensive care unit (ICU) admissions were similar between both groups; however, more patients with sCoV were successfully extubated and successfully discharged from the ICU than patients with COVID-19. The inpatient case fatality rate was significantly higher in patients with COVID-19 compared with patients with sCoV infection.

In the unconditional logistic regression model with generalized estimating equations, patients with COVID-19 presented a significantly increased risk of death compared to patients with sCoV infection (adjusted Odds Ratio [aOR] 3.86, Confidence Interval 1.99–7.49; p < 0.001) (Table 4). We performed three sensitivity analyses. First, using an automated variable selection procedure, we performed a backward stepwise (likelihood ratio) logistic regression to compare our variable selection model based on current evidence of known risk factors associated with viral respiratory infections severity with an automated variable selection model. Covariates with the greatest P-value were progressively removed until only covariates with a P-value less than 0.10 remained in a block with significant improvement of fit compared to the previous block. In this model, COVID-19 remained as a significant predictor of death compared with sCoV infection (aOR 3.42 [1.76–6.63]; p < 0.001). Second, we adjusted the regression model with a propensity score that was calculated from saving the predicted probabilities of a logistic regression with COVID-19 or sCoV infection as dependent variable and age and sex as independent variables, then adjusted the backward selection regression model by including predicted probabilities as a covariate. Additionally, the backward selection regression model was also performed with the logit of the predicted probabilities as a covariate. Lastly, given the lack of a standardized protocol regarding when to order a respiratory multiplex panel by PCR within the Integrated Healthcare System, there is an inherent selection bias towards patients with more severe sCoV infection as physicians tend to order this panel for patients with severe respiratory infections where a precise microbiologic diagnosis is more important. Thus, we performed a subgroup analysis with a model that only included patients admitted to the ICU. Again, COVID-19 carried a significantly greater risk of death compared to sCoV infection (aOR 5.42 [2.08–14.08]; p = 0.001) (Table 4).

**Table 4:** Multivariable regression analysis

Independent variable	Adjusted OR (95% CI)	P-value
Overall population		
COVID-19 (vs. sCoV)	3.86 (1.98–7.49)	< .001
Age	1.02 (0.99–1.03)	.228
Dwelling (LTCF vs. Home)	0.71 (0.34–1.43)	.339
DNR/DNI status	6.2 (2.87–13.36)	< .001
qSOFA score	3.61 (2.40–5.43)	< .001
ICU only		
COVID-19 (vs. sCoV)	5.42 (2.08–14.08)	.001
Age	1.02 (0.98–1.05)	.220
Dwelling (LTCF vs. Home)	0.54 (0.19–1.49)	.236
DNR/DNI status	9.94 (3.11–31.73)	< .001
qSOFA score	1.64 (0.91–2.94)	.096
Backward selection		
COVID-19 (vs. sCoV)^a^	3.42 (1.76–6.63)	< .001
DNR/DNI status	7.74 (4.06–14.74)	< .001
qSOFA score	3.33 (2.29–4.83)	< .001
Sex (male vs. female)	1.94 (1.03–3.66)	.039
Malignancy or mass	2.04 (0.90–4.57)	.085
Severe illness	3.92 (1.53–9.99)	.004

^a^Adjusted OR after propensity score adjustment: 3.511 (95% CI 1.802–6.844); aOR after logit adjustment: 3.511 (95% CI 1.801–6.843)

CI: confidence interval; COVID-19: Coronavirus Disease 2019; DNR/DNI: do-not-resuscitate/do-not-intubate; ICU: intensive care unit; LTCF: long-term care facility; OR: Odds Ratio; qSOFA: quick Sequential Organic Failure Assessment; sCoV: seasonal coronavirus

## Discussion

This retrospective cohort study examined the characteristics and clinical outcomes of hospitalized patients with sCoV infection compared to patients with COVID-19. Patients with COVID-19 presented a higher case fatality rate and an almost fourfold increased risk of death than patients with sCoV. Interestingly, the rates of ICU admission and IMV use were not significantly different. However, more patients with sCoV were extubated and were more likely discharged from the ICU than patients with COVID-19. Seasonal coronaviruses are usually associated with mild upper respiratory illness in adults and are not a considerable public health burden [16]. Though, elderly individuals and immunocompromised hosts can sometimes develop life-threatening bronchiolitis, pneumonia, and even neurological infection (hCoV-OC43) [2]. In one study of community-acquired pneumonia requiring hospitalization among U.S. adults, the incidence of coronaviruses in individuals 80 years of age or older was similar to that of *Streptococcus pneumoniae* [23]. Besides, previous studies have linked common respiratory viruses, including sCoV, with COPD exacerbations, asthma exacerbations, and worsening cardiovascular disease [24–27]. In our cohort, patients admitted with sCoV were found to be initially admitted due to exacerbation of a pre-existing condition, namely heart failure exacerbation and COPD or asthma exacerbation, and later found to have a sCoV infection, where coronaviruses were likely responsible for disease aggravation, as demonstrated by the significantly higher proportions of patients with sCoV infection and underlying cardiovascular disease, obstructive pulmonary disease, and immunodeficiency in comparison to patients with COVID-19. In contrast, most patients with SARS-CoV-2 infection were merely admitted due to COVID-19 and its complications.

The clinical spectrum of hospitalized patients with SARS-CoV-2 infection has been mainly compared to SARS, MERS, and other pandemic viruses [28, 29]; nevertheless, our data shows significant differences with these viruses and important similarities with hospitalized patients with sCoV infection. For instance, although all coronaviruses can affect persons in all age groups, hospitalized patients with COVID-19 and sCoV infection were found to be older (median age 69 and 74 years, respectively). In contrast, previous series reported younger populations affected by SARS and MERS (median age 39 and 56 years, respectively) [30–35]. COVID-19 and MERS affected more male patients, while sCoV and SARS affected predominately female patients. Overall, SARS series reported fewer patients with pre-existing underlying conditions (10 to 30%) [30–32], while in MERS series, 50 to 96% of patients were reported to have at least one underlying condition [33–35]. Similar to MERS series, more than 80% of hospitalized patients with sCoV and COVID-19 had two or more underlying comorbidities in our cohorts. For COVID-19, sCoV, and MERS, the most common presenting symptoms included fever, cough, and shortness of breath, while in SARS series, fever and cough were more prominent relative to shortness of breath [30–35]. Leukopenia on admission was less common in our cohort of patients with sCoV (6.3%) and COVID-19 (9.5%) compared to previous MERS (14–42%) and SARS (25–35%) series [34, 35], whereas lymphopenia rates were similar in patients with sCoV (71.6%), COVID-19 (78.9%), and SARS (68–85%) in comparison to MERS (34%) [35]. As expected, rates of bilateral or multifocal infiltrates at admission were overall higher in patients with COVID-19 (61.6%), SARS (29–45%), and MERS (26–80.3%) than in patients with sCoV infection (30.5%) [30–34]. The rates of ICU admission among patients with sCoV (35.3%) and COVID-19 (32.1%) in our cohorts were higher than in SARS series (20–26%) but lower than in MERS series (78–89%) [30–33, 35]. Overall, the rates of IMV were higher in MERS series (24.5–80%), followed by our cohort of patients with COVID-19 (19.5%), SARS series (13.8–21%), and our cohort of patients with sCoV infection (14.2%) [30–35]. Case fatality rates were higher in series of hospitalized patients with MERS (20.4–65%), followed by our cohort of hospitalized patients with COVID-19 (34.7%), SARS series (3.6–13.6%), and our cohort of hospitalized patients with sCoV infection (11.6%) [30–35]. Considering all patients, including outpatients and inpatients, the estimated case-fatality rate of COVID-19 is around 1–3%, 9.5–15% for SARS, and 34.4% for MERS. The overall case-fatality rate for seasonal coronaviruses is not well described [28, 29]. However, using data from the Underlying Cause of Death tool in the CDC Wide-ranging ONline Data for Epidemiologic Research (CDC WONDER) Online Database and the National Respiratory and Enteric Virus Surveillance System (NREVS), we estimated a rough case fatality rate of 0.0027% (108 deaths from unspecified coronavirus illness reported between the years 2014–2017 in the CDC WONDER Online Database and 39 588 cases of HCoV reported to the NREVSS during the same period) [5, 36].

Compared to other respiratory pathogens other than coronaviruses, COVID-19 shares some similarities but also has a unique disease spectrum. In a study by Shah et al., similarly to our results, most comorbidities, medications, symptoms, vital signs, laboratories, treatments, and outcomes did not differ between patients with and without COVID-19. However, patients with COVID-19 were more likely to be admitted to the hospital (79% vs. 56%, p = 0.014), have more extended hospitalizations (median 10.7 days vs. 4.7 days, p < 0.001), and develop ARDS (23% vs. 3%, p < 0.001), and were unlikely to have co-existent viral infections compared with patients with an acute respiratory illness different that COVID-19 [11]. Furthermore, Spieza et al. showed that patients with COVID-19 pneumonia had significantly shorter clot formation time and higher maximum clot firmness (P < 0.01 and P < 0.05, respectively) than patients with non-COVID-19 pneumonia [12].

In a systematic review that compared COVID-19 to influenza, comorbidities such as cardiovascular diseases, diabetes, and obesity were significantly higher in COVID-19 patients. In contrast, pulmonary diseases and immunocompromised conditions were significantly more common in influenza patients, similar to our population with sCoV infection. Neurologic symptoms and diarrhea were statistically more frequent in COVID-19 patients compared to influenza patients, reminiscent of our cohort of COVID-19 patients. Ground-grass opacities and a peripheral distribution were more common in COVID-19 patients than in influenza patients, where consolidations and linear opacities were described instead. In comparison, our patient population with COVID-19 also most commonly presented diffuse opacities with bilateral distribution compared with patients sCoV infection. Lastly, COVID-19 patients were found to have significantly worse outcomes than influenza patients: More often transferred to intensive care unit with a higher rate of mortality [13]. The severity of COVID-19 compared to influenza was demonstrated again in a study by Talbot et al., where patients with COVID-19 showed greater severity and complications, including more ICU admissions (aOR 5.3, 95% CI 11.6–20.3), ventilator use (aOR 15.6, 95% CI 10.7–22.8), seven additional days of hospital stay in those discharged alive, and death during hospitalization (aOR 19.8, 95% CI–12.0, 32.7) [14].

With the expansion of SARS-CoV-2 worldwide, the emergence of new, more transmissible variants [37, 38], and the variable effectiveness of current vaccines against those variants [39], there is little hope for eliminating the virus from the human population. Unlike SARS-CoV and MERS-CoV, which were locally contained, SARS-CoV-2 will likely transition to endemicity and continued circulation with the other sCoVs [16]. Seasonal coronaviruses have annual circulation peaks in the winter months in the U.S., and individual species show variable circulation from year to year [5]. Recent data from the NREVSS showed that during the 2019–20 winter season, HCoV-HKU1 was the most common sCoV circulating in the U.S., followed by HCoV-NL63. In comparison, during the 2020–21 winter season, HCoV-OC43 was the most common sCoV circulating in the U.S., again followed by HCoV-NL63 [40]. Our cohort encompassing nine years, the most common isolated sCoV was HCoV-OC42, followed by HCoV-HKU1. Although it is not clear whether COVID-19 will become a chronic seasonal disease, numerous epidemiological studies and models have explored the relationship between COVID-19 transmission and meteorological factors. These models have shown that infectivity of SARS-CoV-2 and mortality of COVID-19 are more substantial in colder climates and that COVID-19 seasonality is more pronounced at higher latitudes where larger seasonal amplitudes of environmental indicators are observed [15, 41], supporting the circulation of SARS-CoV-2 as a seasonal respiratory pathogen.

This study has several limitations. As mentioned before, one of the most significant limitations is the selection bias associated with the inpatient use of the respiratory multiplex panel by PCR. Since its availability and up to the writing of this manuscript, there is no formal protocol in place within the Integrated Health System regarding when to order this test. Physicians can order the panel at their discretion. In consequence, there may be a selection bias towards patients with more severe disease, whereas patients with less severe disease were omitted. We tried to address this issue with a sensitivity analysis, including only critically ill patients. Another significant limitation is the fact that the data of the COVID-19 population analyzed in this study were obtained during the initial wild-type (Wuhan-Hu-1) phase in the United States and before the emergence of variants of concern that later replaced the wild-type virus, namely Alpha, Delta, and Omicron, that have been shown to have different biological, epidemiological and clinical characteristics [42, 43]. This was a retrospective cohort study, and clinical data were retrospectively collected through electronic medical records and manual chart review. Therefore, a degree of inter-rater variability is expected. Second, the present study was observational and included populations of patients distributed at different points in time; thus, unknown risk factors and bias might have been unequally distributed between the two groups in the analysis. The subjects with COVID-19 included for analysis encompass a series of consecutively admitted patients early in the pandemic before using steroids as the standard of care and the development of standardized, evidence-based management guidelines, and widespread availability of COVID-19 vaccines, which have shown to have a significant impact on morbidity and mortality. On the other hand, the cohort of subjects with sCoV infection included patients from a period of 9 years, during which progress in medical knowledge and patient care are expected; hence, the crude case-fatality ratio must be taken with caution. Finally, the analyzed population was limited to one Integrated-Delivery Health system in the Chicago metropolitan area and may have limited external generalizability.

## Conclusions

In conclusion, the clinical spectrum of hospitalized patients with COVID-19 is more similar to SARS and MERS in terms of illness severity and case-fatality rate than hospitalized patients with sCoV infection. However, the demographics and baseline characteristics of patients hospitalized with COVID-19 and sCoV infection are more similar, affecting older populations with many underlying conditions, making it difficult to distinguish both entities solely on a clinical basis. Thus, should SARS-CoV-2 transition into an endemic virus after the pandemic, clinical findings alone may not help confirm or exclude the diagnosis of COVID-19 during high acute respiratory illness seasons. With the availability of specific COVID-19 therapies and infection prevention protocols, the respiratory multiplex panel by PCR that includes SARS-CoV-2 in conjunction with local epidemiological data may be a valuable tool to assist clinicians with management decisions.

## Data Availability

The data and materials used to support the findings of this study are available from the corresponding author upon reasonable request. The local IRB committee prohibits the release of the dataset without protocol amendments.

## Abbreviations

AKI: Acute kidney injury
ARDS: Acute respiratory distress syndrome
CDC WONDER: CDC Wide-ranging ONline Data for Epidemiologic Research
COVID-19: Coronavirus disease 2019
DNR/DNI: Do-not-resuscitate/do-not-intubate
HCoV: Human Coronavirus
IQR: Interquartile range
IVM: Invasive mechanical ventilation
LTCF: Long-term care facility
MERS: Middle East respiratory syndrome coronavirus
NREVSS: National Respiratory Enteric Virus Surveillance System
qSOFA: Quick Sequential Organ Failure Assessment
sCoV: Seasonal coronavirus
SARS: Severe acute respiratory syndrome
