# Correction: Comparison of clinical characteristics and outcomes of hospitalized patients with seasonal coronavirus Infection and COVID-19: a retrospective cohort study

**DOI:** 10.1186/s12879-023-08782-z

**Published:** 2024-01-15

**Authors:** Guillermo Rodriguez-Nava, Goar Egoryan, Tianyu Dong, Qishuo Zhang, Elise Hyser, Bidhya Poudel, Maria Adriana Yanez-Bello, Daniela Patricia Trelles-Garcia, Chul Won Chung, Bimatshu Pyakuryal, Taraz Imani-Ramos, Valeria Patricia Trelles-Garcia, Daniel Sebastian Bustamante-Soliz, Jonathan J. Stake

**Affiliations:** 1 Department of Internal Medicine, AMITA Health Saint Francis Hospital, 355 Ridge Ave, Evanston, IL 60202 USA; 2 Department of Internal Medicine, AMITA Health Saint Joseph Hospital, Chicago, IL USA; 3 Department of Internal Medicine, John H. Stroger Jr. Hospital of Cook County, Chicago, IL USA; 4 Facultad de Ciencias Medicas de La Universidad de Cuenca, Cuenca, Ecuador; 5 Department of Infection Prevention, AMITA Health Saint Francis Hospital, Evanston, IL USA

**BMC Infectious Diseases (2022) 22:618**

10.1186/s12879-022-07555-4

The original publication of this article contained 2 incorrect references to “OC93” which should have been “OC43”. The original article has been updated.

